# Certificates of confidentiality: privileging research data

**DOI:** 10.1093/jlb/lsae003

**Published:** 2024-02-22

**Authors:** Leslie E Wolf, Natalie Ram, Jorge Contreras, Laura M Beskow

**Affiliations:** Georgia State University College of Law, 85 Park Place, Atlanta, GA, 30303, United States; University of Maryland Francis King Carey School of Law, 500 W. Baltimore Street, Baltimore, MD 21201, United States; University of Utah S.J. Quinney College of Law, 383 South University Street, Salt Lake City, UT 84112-0730, United States; Vanderbilt University School of Medicine, 2525 West End Avenue, Nashville, TN 37203, United States

**Keywords:** protection of research subjects, confidentiality, ethics, law

## Abstract

With the Supreme Court’s decision in *Dobbs*, reproductive research now joins other sensitive research topics that present legal risks to research participants, underscoring the role of Certificates in protecting them. Yet, stakeholders question whether Certificates will hold up in court. In this article, we describe the essential arguments supporting Congress’s regulation of biomedical research and, thus, Certificates, under its authority to regulate interstate commerce. Our analysis should reassure researchers and Institutional review boards who rely on Certificates to protect the confidentiality of research participants’ data. We conclude with recommendations for stakeholders based on our analysis.

In the wake of the Supreme Court’s decision in *Dobbs*,^1^ numerous aspects of reproductive health have become criminalized,^2^ making research in this area risky for both patients and researchers.^3^ Such risks are not unique to reproductive health research. Investigators conducting research on substance abuse and HIV/AIDS, for example, have grappled with how to conduct important public health research ethically, when the data they collect could be used against participants and researchers in criminal, civil, or disciplinary actions or give rise to other harms were the data revealed outside the research.^4^ Nor are these risks theoretical. Research data have been sought, for example, by prosecutors,^5^ criminal defendants,^6^ civil litigants,^7^ non-governmental organizations,^8^ and government agencies.^9^ Police have mined consumer genetics databases, some of which also conduct research, hundreds of times since the ‘Golden State Killer’ case to investigate crimes.^10^ Police have also accessed DNA from newborn screening programs, also often used in research, for investigative purposes in at least five criminal cases.^11^

To meet their ethical obligations to minimize risks to participants and protect confidentiality,^12^ researchers collecting research data that could be of interest in legal proceedings have relied on the Certificate of Confidentiality, a federal statutory device that protects identifiable, sensitive biomedical research data from compelled disclosure.^13^ Specifically, if a law enforcement officer, prosecutor, legislator, civil litigant, or other party seek to compel disclosure of identifiable information about a research participant through a subpoena or warrant, the Certificate prohibits the researcher from making the disclosure and bars the use of that information as evidence.^14^

Importantly, following amendments made by the 21st Century Cures Act, the Certificate statute entitles all federally funded research projects to its protections, and researchers not federally funded are eligible to apply for one.^15^ The National Institutes of Health (NIH) began to issue Certificates automatically to the research it funds as part of the terms and conditions of the grant.^16^ Accordingly, the protections Certificates afford now apply to vast amounts of health-related research data.

Certificates are essential to allowing certain types of research to proceed.^17^ Institutional review boards (IRBs) have often made approval of a study contingent on receipt of a Certificate to ensure that participants are adequately protected.^18^ At the same time, researchers, IRBs, and legal counsel have raised questions about the legal effect of Certificates and whether they can effectively shield participant data.^19^ Given these concerns, when faced with a legal demand, counsel have often used other means to protect research data from disclosure to avoid testing whether Certificates would hold up in court.^20^ These lingering concerns led us to explore, in other work, the constitutional underpinnings of Certificates.^21^ Here, we describe the essential arguments supporting Congress’s regulation of health-related research (of which Certificates are a subpart) under its authority to regulate interstate commerce. Our analysis should reassure researchers and IRBs who rely on Certificates to protect the confidentiality of research participants’ data. Coupled with prior work, this analysis also provides researchers, IRBs, and legal counsel with potential legal defenses if they are subject to a legal demand for data protected by a Certificate. We conclude with recommendations for stakeholders based on our analysis.

## I. CERTIFICATES OF CONFIDENTIALITY—BACKGROUND

### I.A. Congressional Authorization of Certificates

Congress first authorized Certificates under the Comprehensive Drug Abuse Prevention and Control Act of 1970.^22^ That Act sought ‘to deal in a comprehensive fashion with the growing menace of drug abuse’ with focus on research, rehabilitation, and punishment.^23^ In hearings before the Act’s passage, Congress heard about the critical need for research on drug abuse, as well as researchers’ and potential participants’ fears that this research could place them in legal jeopardy.^24^ Researchers also testified that they would risk being found in contempt of court and jailed rather than reveal participant identities.^25^ Certificates, as originally envisioned, sought to promote the study of narcotics and addiction by insulating individually identifiable research data against compelled disclosure in any ‘Federal, State, or local, civil, criminal, administrative, legislative, or other proceeding’.^26^

Since 1970, Congress has modified the statute authorizing Certificates multiple times. In 1974, it added protections for mental health and alcohol research.^26^ A more significant change came in 1988, when the statute was amended to extend to ‘biomedical, behavioral, clinical, or other research in which identifiable, sensitive information is collected (including research on mental health and on the use and effect of alcohol and other psychoactive drugs)’.^27^ (For convenience, we hereinafter refer to types of research to which Certificates may apply collectively as ‘biomedical research’. This reference is consistent with the full range of research and research methodologies NIH and other federal agencies fund to ‘enhance health, lengthen, life, and reduce illness’.)^28^ This amendment resulted in Certificates’ protections applying to all research involving sensitive, identifiable information rather than being limited to a specific research area.

In 2016, the 21st Century Cures Act implemented multiple, substantive revisions to the legal framework surrounding Certificates.^29^ While the categories of protectable research remained unchanged, the Act made the issuance of Certificates mandatory for all federally funded research, expanded the disclosure prohibition beyond identifiers, removed language permitting voluntary disclosures (enacted alongside expanded exceptions to the disclosure prohibition), made information protected by Certificates inadmissible as evidence in legal proceedings of all kinds, and defined identifiability.^30^

Under the current statute, Certificates are available to ‘biomedical, behavioral, clinical, or other research in which identifiable, sensitive research is collected (including research on mental health and on the use and effect of alcohol and other psychoactive drugs)’.^31^ The Secretary of Health and Human Services (HHS) ‘shall’ issue a Certificate to research that is funded by the federal government and ‘may’ issue Certificates to non-federally funded research.^32^ The protections extend not only to the participant’s name but also to ‘any [] information, document, or biospecimens that contains identifiable, sensitive information about such an individual and that was created or compiled for the purpose of research’.^33^ Researchers who receive a Certificate ‘shall not’ disclose identifiable, sensitive information ‘in any Federal, State, or local, civil, criminal, administrative, legislative, or other proceeding, except as authorized in the statute’.^34^ In addition, the protected information is ‘immune from legal process’, and absent consent of the individual whose data is at issue ‘shall not…be admissible as evidence or used for any other purpose in any action, suit, or other judicial, legislative, or administrative proceeding’.^35^ These protections apply to ‘all copies…for perpetuity’.^36^

The Certificate statute does not specify the penalties for breach of its provisions. Because a Certificate automatically issues and becomes part of the terms and conditions of applicable NIH-funded grants, disclosures that do not comply with the Certificate statute may give rise to penalties for non-compliance or could be used as evidence of breach in other legal actions.^37^

### I.B. Certificates in Action

There are relatively few published legal decisions involving Certificates.^38^ The first and best-known case involving a Certificate, *People v Newman*,^39^ is powerful factually. Dr Newman was found to be in contempt of court after asserting that a Certificate justified his refusal to produce photographs of a methadone clinics’ patients to law enforcement officers who wanted to use them to identify a homicide suspect.^40^ The Court ultimately determined that the Certificate protected the photographs from disclosure. But the court’s legal analysis of the Certificate’s protections is sparse.^41^

The only other case directly interpreting a Certificate confirmed that data can be disclosed with the consent of the individual whose data are at issue.^42^ Often, however, courts appear to skirt the Certificate as a legal tool of protection. The Certificate’s protections were asserted in *North Carolina v Bradley* when a criminal defendant sought access to research records to impeach a prosecution witness. Both the trial court and the appellate court denied the defendant’s request on the ground that the records were immaterial to the defense. Neither court considered whether the Certificate would prohibit disclosure had the records been material to the defense.^43^ Moreover, the trial court ordered disclosure of the research records to defense counsel and state’s counsel (albeit under a protective order) to allow for purposes of the appeal, rather than conducting an *in camera* review.^44^ While the appellate court ultimately rescinded the defendant’s access to the documents, from the researcher’s perspective, ‘confidentiality had already been compromised,’ despite assurances provided to participants.^45^

Research data are of interest in civil cases as well as criminal cases. There are multiple tort cases involving exposure to drugs or chemicals in the environment, in which redacted research data were disclosed, either by agreement or court order. These disclosures were often accompanied by protective orders or agreements to preserve confidentiality.^46^ In contrast, one juvenile court case required disclosure of identifiable research data concerning four abused or neglected children to the Connecticut Department of Families and Children.^47^ Arguably, the disclosure was ordered to protect the children, but the Department had already followed up on researcher reports and secured both physical and legal custody of the children. In another civil case, a non-profit organization sought data from a study commissioned to evaluate racial fairness in sentencing. Kentucky’s Attorney General determined that the Certificate precluded production of data because, although the data were not identifying by themselves, they would be identifying if combined with publicly available information.^48^ There are other cases that mention Certificates, but these often seem to reflect confusion by parties, attorneys, or judges about when a Certificate is relevant within research, as well as whether it can apply outside of research.^49^

Interviews with legal counsel expand on the experiences reflected in the cases described above.^50^ Counsel have expressed concerns about losing a Certificate case and thereby undermining the protections of Certificates and harming the research community.^51^ While explaining the Certificate’s protections to a requester of research data has often been sufficient to thwart a legal demand, if the requester persisted, counsel would direct the requester to other sources of the data (eg medical records) or produce redacted data.^52^ If required to assert a Certificate in court, counsel often relied on legal arguments other than the Certificates, rather than risk a ruling that the Certificate did not protect against disclosure.^53^

Given legal counsels’ concerns about the legal effect of Certificates—concerns not alleviated by the case law, it is important to appreciate that the Certificate is awarded to the institution, not to the principal investigator.^54^ It is institutional legal counsel who will ultimately determine what strategy to employ in the face of a legal demand. Investigators and legal counsel may view demands for data differently, with investigators focused on their research and participants and counsel focused more broadly on institutional interests. Neither group is likely to be particularly knowledgeable about Certificates nor to have experienced a legal demand for data.^55^ With this context in mind, we now turn to our analysis of Congress’s authority to regulate research and to create the protections afforded by Certificates.

## II. CONGRESSIONAL AUTHORITY TO REGULATE RESEARCH UNDER THE COMMERCE CLAUSE

Congress’s powers are limited to those enumerated in the US Constitution; all other powers are reserved to the states.^56^ Of Congress’s enumerated powers, two are most relevant for our purposes—the power to regulate commerce ‘among the several states’ and the power to tax and spend for the general welfare. Each of these is coupled with the power ‘to make all laws which shall be necessary and proper for carrying into execution’ those powers.^57^

### II.A. Commerce Clause Authority—Background

The Supreme Court has explained that, pursuant to the Commerce Clause, Congress may regulate: (i) ‘the channels of interstate commerce’, (ii) ‘the instrumentalities of interstate commerce, or persons or things in interstate commerce’, and (iii) ‘those activities having a substantial relation to interstate commerce, ie those activities that substantially affect interstate commerce’.^58^

The Court’s decisions have historically recognized several key guardrails for identifying the Commerce Clause’s outer limits. First, Congress must have had a rational basis to conclude that the regulated activity ‘*substantially* affect[s]’, and not merely ‘affect[s]’, interstate commerce.^59^ Second, regulated activity must be ‘commercial’ or ‘economic’ in nature. The Court has rejected Congress’s efforts to regulate when it has found that the underlying activity was not itself ‘economic’ in nature or the link between the regulated activity and interstate commerce was ‘attenuated’.^60^

Even so, the scope of ‘economic’ activity is quite broad.^61^ Moreover, the Commerce Clause does not ‘require[] Congress to legislate with scientific exactitude. When Congress decides that the total incidence of a practice poses a threat to a national market, it may regulate the entire class’.^62^ Thus, Congress may regulate under the Commerce Clause when the activity being regulated is itself broadly ‘economic’ and its relationship to the interstate economy is reasonably straightforward.

Third, congressional action under the Commerce Clause must regulate commercial or economic ‘activity.’ That is, while Congress may regulate existing commercial or economic transactions, processes, and the like, it may not compel actors to engage in such activity.^63^ Finally, in analyzing disputes regarding Congress’s enumerated powers, the Court has recognized that these powers are strengthened by Congress’s authority to ‘make all Laws which shall be necessary and proper for carrying into Execution the foregoing Powers’^64^—the so-called Necessary and Proper Clause.

With this understanding of Congress’s Commerce Clause power, we now consider how biomedical research fits within this legal framework. In what follows, we show that Congress’s authorization of Certificates is a lawful exercise of its Commerce Clause authority because Certificates are part and parcel of Congress’s regulation of biomedical research, which is decidedly interstate economic activity. Moreover, biomedical research, research data itself, and research results are ‘things in interstate commerce’.^65^ Taken together, the activities and products of research that Certificates of Confidentiality seek to regulate ‘substantially affect interstate commerce’.

### II.B. Biomedical Research as Interstate Commerce

The purpose of biomedical research, broadly stated, is to develop generalizable knowledge to improve the health and well-being of the population.^66^ Federal law requires clinical research demonstrating safety and efficacy of drugs, devices, and biologics to receive marketing approval by the Food and Drug Administration (FDA).^67^ Research results may also inform clinical practice, especially as the medical profession has embraced evidence-based medicine and, more recently, learning health systems to improve patient care.^68^ Hence, biomedical research is essential to the functioning of the pharmaceutical industry and the health care system, which together comprise one of the largest sectors of the US economy.^69^ Needless to say, the impact of biomedical research extends to every state.^70^

Hundreds of billions of dollars are spent each year to support the biomedical research enterprise.^71^ It is not easy to determine exactly how much is spent on human subjects research. But with its annual budget ($41.7 billion in 2020),^72^ NIH provides grants to ‘more than 300,000 researchers at more than 2500 universities, medical schools, and other research institutions in every state’ and supports ‘nearly, 6000 scientists in its own laboratories’.^73^ In addition to NIH, other federal agencies support and conduct human subjects research.^74^ For example, the Congressionally Directed Medical Research Program within the US Army alone has annual appropriations that exceed $1 billion.^75^ PhRMA, a trade group representing US pharmaceutical companies, estimates that in 2020, the biopharmaceutical industry invested over $90 billion in research and development.^76^ The biopharmaceutical industry employs hundreds of thousands of individuals across all 50 states.^77^ Expenditures on FDA-approved drugs, devices, and biologics are in excess of $650 billion.^78^ With total expenditures on health care exceeding $4 *trillion* in 2020,^79^ the economic impact of research soars as it enters medical practice.

Moreover, the conduct of biomedical research itself has become increasingly multi-site and multi-state.^80^ With new mandates for single IRB review for collaborative research,^81^ research oversight also often occurs across state lines.^82^ Research results similarly cross state lines through publications. For example, the *Journal of the American Medical Association (JAMA)* is the most widely circulated medical journal with over 2 million recipients worldwide and over 57 million annual visits to its website.^83^

### II.C. Congressional Regulation of Biomedical Research

Congress has a long history of legislative action supporting, facilitating, and overseeing biomedical research. In 1887, Congress authorized funding of a laboratory that would eventually evolve into the Public Health Service and the NIH.^84^ Congress’s involvement in human protections oversight came through its enactment of the National Research Act in 1974.^85^ One of the stated goals of that Act was to ‘maintain[] a superior *national* program of research into the physical and mental disease and impairment of man’ (emphasis added).^86^ The impetus for the National Research Act came from the Associated Press’s reporting in 1972 of the abuses of African American participants in the infamous Tuskegee Syphilis Study.^87^ Responding to the resulting public outcry, the Act established the National Commission for the Protection of Human Subjects of Biomedical and Behavioral Research.^88^ Among its multiple reports is the 1979 Belmont Report that established the framework for the ethical conduct of human subjects research that is still followed today.^89^ Its principles were incorporated into the HHS and the FDA 1981 regulations governing human subjects research, promulgated as directed by Congress.^90^

Congressional acts also created and expanded the authority of the FDA. These statutes have, among other things, extended federal oversight over human subjects in research conducted to support the approval of drugs, medical devices, and biologics,^91^ as well as overseeing that approval process, which requires evidence of safety and efficacy.

In sum, research dollars, researchers, research projects, research data, research results, and their resulting products and practices all cross state lines with increasing frequency and regularity. Given the scale of investment in and reach of each of these, it is apt to describe them as ‘persons or things’ moving in ‘interstate commerce’.^92^

### II.D. Certificates as an Integral Part of Biomedical Research

Certificates regulate access to and protect identifiable, sensitive information about research participants.^93^ These data exist or are collated only by virtue of research activity. Congress reasonably concluded that regulating access to such data was necessary to facilitate more and better quality research. Accordingly, insofar as research data and results constitute ‘things’ in ‘interstate commerce’, Congress’s authorization of Certificates fits well within that body’s regulation of biomedical research under its Commerce Clause authority.^94^

Congress’s regulation of biomedical research, including its creation of Certificates, is further supported by the third category of activity that Congress may regulate under the Commerce Clause—‘activities that substantially affect interstate commerce’.^95^ In light of the scale of investment, employment, and resulting spending, there can be little dispute that biomedical research and its results ‘substantially affect interstate commerce’. Certificates, in turn, are bound up with the production, analysis, dissemination, and translation of biomedical research. For NIH-funded researchers, a Certificate is now a standard term and condition in a grant award.^96^ The Certificate’s protections, in other words, are integral to the research itself. Moreover, disclosure of identifiable, sensitive research data could, ‘through repetition elsewhere’, profoundly affect biomedical research, which is a part of interstate commerce.^97^ Indeed, Congress enacted the Certificate statute because it concluded that shielding access to research data was essential to facilitating beneficial biomedical research. Thus, there is a close connection between regulating biomedical research and limiting the disclosure and use of research data, rather than an ‘attenuated’ one, and thus is well within Congress’s authority.^98^

This is not a case in which Congress has ‘use[d] a relatively trivial impact on commerce as an excuse for broad general regulation of state or private activities’.^99^ Rather, as described above, biomedical research and the dissemination and translation of its results implicate traditional commerce clause activities. Nor is Congress’s regulation of biomedical research—or even its regulation of the disclosure of data that is collated or produced through that research—novel. The Supreme Court has recently emphasized that ‘sometimes “the most telling indication of [a] severe constitutional problem…is the lack of historical precedent” for Congress’s action.’^100^ But Certificates are firmly rooted in Congress’s past practice. Certificates were first enacted more than half a century ago, with multiple amendments since. And as discussed in more detail below, Congress has passed other laws that protect information from disclosure.^101^

### II.E. Protecting Privacy and Privilege through the Commerce Clause

While our analysis demonstrates that Congress’s acts to establish and amend Certificates fit within its Commerce Clause authority, the legality of its interference with state powers by prohibiting disclosure and use in state ‘judicial, legislative, or administrative proceeding[s]’^102^ merits consideration. In general, the law of privileges, such as attorney–client and physician–patient privilege, is a matter of state common law doctrine.^103^ Underscoring this principle, Federal Rule of Evidence 501, ‘preserve[s] the application of state privilege law in federal courts’.^104^ But Congress nonetheless retains the power to create privileges by statute.^105^

Privileges can be extraordinarily powerful. Rebecca Wexler recently explained that privileges are unique in ‘their breadth, power, and extraordinary costs’,^106^ observing that they ‘apply to every stage of a case, from investigations by law enforcement or criminal defense counsel, to grand jury proceedings, to pretrial, trial, and postconviction proceedings’.^107^ Moreover, ‘[p]rivileges block not merely the admissibility of evidence in court but also litigants’ ability to compel the production of information for their own review. Privileges can shield information from warrants, subpoenas, and discovery orders.’^108^ But privileges also impose significant costs, as they ‘impede the search for the truth’.^109^

Given these features, the Supreme Court has been reluctant to recognize federal statutory privileges.^110^ The Court has explained that ‘[a]statute granting a privilege is to be strictly construed so as “to avoid a construction that would suppress otherwise competent evidence.”’^111^ A pair of Supreme Court cases concerning the Census Act are instructive in understanding when a statute creates a privilege, with stronger obligations than those imposed by mere confidentiality protections. In the first case, the Court found that a section of the Census Act that barred the Commerce Department from using, publishing or permitting anyone to examine census reports merely created confidentiality protections, not a true privilege.^112^ In response, Congress amended the Census Act to state that certain census reports ‘shall be immune from legal process, and shall not, without the consent of the individual or establishment concerned, be admitted as evidence or used for any purpose in any action, suit, or other judicial or administrative proceeding’.^113^ In the second case, the Supreme Court rejected efforts by two localities seeking access to census data, recognizing that the amended Census Act created a statutory privilege for the data sought,^114^ and that Congress had the authority to enact this privilege.^115^

Similarly, in *Pierce County v Guillen*, a unanimous Supreme Court upheld Congress’s authority, pursuant to its Commerce Clause power, to enact a privilege enforceable in state and federal courts alike.^116^  *Guillen* considered a federal statute that required state and local governments to survey ‘hazardous locations’ on their roads in order to qualify for federal funds to ‘improve the most dangerous sections of their roads’.^117^ But fears that this information could be used against them in litigation reduced states’ and local governments’ willingness to collect it.^118^ Accordingly, Congress amended the statute to state that information ‘compiled or collected’ to qualify for such funding ‘shall not be subject to discovery or admitted into evidence in a Federal or State court proceeding or considered for other purposes in any action for damages arising from any occurrence at a location mentioned or addressed’ in the compiled or collected information.^119^ The Court in *Guillen* held that this language sufficed to establish a privilege.

Consistent with these cases, courts and scholars have identified key features that identify a statutory privilege. The clearest examples of statutory language creating a privilege are provisions stating that information is ‘immune from legal process’, ‘shall not…be admitted as evidence’, or ‘shall not be subject to discovery’.^120^ This form of language is not uncommon across the US Code,^121^ and courts have repeatedly contrasted less explicit language with these formulations to explain why the alternate wording does not create a true privilege.^122^

The Certificate statute utilizes that every one of the formulations courts have recognized as indicating a statutory privilege—and then some. The Certificate statute makes sensitive, identifiable research data ‘immune from the legal process’.^123^ Furthermore, the statute provides that these data ‘shall not, without the consent of the individual to whom the information pertains, be admissible as evidence or used for any purpose in any action, suit, or other judicial, legislative, or administrative proceeding’.^124^ The Certificate statute also contains additional provisions emphasizing the strength and scope of its protections. Persons to whom a certificate is issued are instructed that they ‘shall not, in any Federal, State, or local civil, criminal, administrative, legislative, or other proceeding, disclose or provide the name of such individual or any such information, document, or biospecimen that contains identifiable, sensitive information about the individual and that was created or compiled for purposes of the research, except’ with the consent of the research participant whose information is at issue.^125^ These protections apply to all copies of the identifiable, sensitive research data ‘for perpetuity’.^126^ With the totality of this statutory language, there can be little doubt that Certificates establish a statutory privilege.

Certificates are arguably broader in their reach and scope than the privilege upheld by the Supreme Court in *Guillen*, as well as many other statutory privileges. The Certificate statute, after all, applies not only to federal and state judicial proceedings but also extends to administrative and legislative proceedings. But these features ought not undermine the Certificate’s constitutionality under the Commerce Clause. In *Guillen*, the Supreme Court established that Congress has the authority to privilege information in federal and state civil proceedings.^127^ As to other proceedings, none is unprecedented in federal law, or indeed in Commerce Clause-related legislation. Congress has elsewhere privileged relevant information from disclosure in criminal proceedings^128^ and legislative proceedings.^129^ In addition, Congress, exercising its Commerce Clause power, has enacted data regulations that bind both government and private actors.^130^

In sum, neither Congress’s creation of a statutory privilege nor that regulation’s potential impact on state and local government entities runs afoul of the Commerce Clause. The Supreme Court blessed Congress’s authority to create a statutory privilege associated with a Commerce Clause-related program in *Guillen*. And Congress has not infrequently acted pursuant to the Commerce Clause to regulate even state and local government access to sensitive data in private hands. These statutes have either survived constitutional scrutiny under the Commerce Clause or have not been subject to such challenge at all. Insofar as Congress may regulate biomedical research as ‘interstate commerce’, a Certificate should constitutionally be capable of privileging access to the data that biomedical research produces.

### II.F. Certificates and Comprehensive Regulatory Schemes

The Supreme Court has often recognized that Congress may regulate comprehensively and, when it does so, such ‘comprehensive regulatory statutes may be validly applied to local conduct that does not, when viewed in isolation, have a significant impact on interstate commerce’.^131^ For example, in *Gonzales v Raich*, the Supreme Court upheld the application of the Controlled Substances Act to the ‘intrastate, noncommercial cultivation, possession, and use of marijuana’.^132^ In reaching this conclusion, the Court emphasized that the Controlled Substances Act, which was ‘enacted in 1970 as part of the Comprehensive Drug Abuse Prevention and Control Act, was a lengthy and detailed statute creating a comprehensive framework for regulating the production, distribution, and possession of five classes of “controlled substances.”’^133^ Concurring in *Raich*, Justice Scalia further explained that, in his view, ‘Congress may regulate even noneconomic local activity if that regulation is a necessary part of a more general regulation of interstate commerce’.^134^

The comprehensive statute at issue in *Raich* is significant. As the Court in *Raich* observed, all parties to the case agreed ‘that passage of the CSA, as part of the Comprehensive Drug Abuse Prevention and Control Act, was well within Congress’s commerce power’.^135^ The original Certificate statute was enacted as part of this same comprehensive legislative act.^136^ As Congress has broadened the scope of the Certificate statute over the decades, it has done so as part of other comprehensive regulatory efforts, most recently as part of the 21st Century Cures Act.^137^ Thus, the Court’s analysis of comprehensive regulatory schemes provides additional support for the constitutionality of the Certificate statute under the Commerce Clause.^138^

### II.G. Spending Clause Authority

Finally, for research that is funded or conducted by the federal government, the Spending Clause provides additional support for Certificates.^139^ The Supreme Court has recognized Congress’s authority to impose conditions on the receipt of federal funds and has generally deferred to Congress’s judgment regarding what conditions may be imposed. The Court has rejected conditions that do not afford the recipient true choice, but our analysis suggests that this concern does not apply to Certificates. Of course, the Spending Clause authority does not extend to Certificates that cover privately funded research.

As we demonstrate above, Certificates stand on firm constitutional footing under the Commerce Clause. Biomedical research activities (and the Certificate protections they include) encompass ‘things in interstate commerce’ or at least ‘activities that substantially affect interstate commerce’.^140^ The strong and broad protection that Certificates provide—shielding relevant research data from legal process across a broad range of proceedings—does not undermine that conclusion. Congress has ample authority to regulate access to sensitive data and even to enact statutory privileges, including under the Commerce Clause, and courts have validated Congress’s exercise of that power where the data related to interstate commercial activity. That authority is further supported by the Certificate’s status as a consistent fixture in comprehensive regulatory schemes, particularly those focused on biomedical research and innovation.

## III. EXCEPTIONAL EXCEPTIONS

Notwithstanding the foregoing, there may be limited circumstances under which Certificate protections may be required to give way to legal demands for data, which we address in this section.

### III.A. Anti-Commandeering Implications

The anti-commandeering doctrine prohibits the federal government from controlling—or ‘commandeering’—state governments in ways that infringe on state powers. Specifically, it prevents the federal government from ‘imposing targeted, affirmative, coercive duties upon state legislators or executive officials’.^141^

In a series of decisions, the US Supreme Court has illuminated the difference between permissible and impermissible effects of federal regulation on state government. It has held that Congress may not compel state law enforcement officials to conduct background checks under the Brady Act^142^ and that federal law ‘prohibiting states from “authorizing” sports gambling unconstitutionally “commandeered” the authority of state legislatures’.^143^ In contrast, the Court has also held that Congress *may* prevent states from selling information that citizens provide to state Departments of Motor Vehicles to obtain drivers’ licenses.^144^ In the latter case, the Court concluded that individuals’ personal information should be considered an ‘article of commerce’, and thus, Congress could regulate its sale under the Commerce Clause.^145^

Although a Certificate dispute could arise in a state legislative or administrative proceeding, thus implicating the anti-commandeering doctrine, we anticipate that most (if not all) disputes over Certificate protections will arise in criminal or civil proceedings.^146^ The Supreme Court has not decided whether the creation and enforcement of a federal statutory privilege in a state proceeding runs afoul of the anti-commandeering principle.^147^ However, it has explicitly stated that it is permissible to require state courts to enforce federal law under the Supremacy Clause.^148^

### III.B. Criminal Defense Rights

Those relying on Certificates have identified the conflict between the Certificate’s statutory privilege and a criminal defendant’s constitutional right to present a complete defense as an area of concern.^149^ In a series of cases, the Supreme Court has elaborated from the Sixth Amendment and the Due Process Clause what amounts to a constitutional right to present a defense.^150^ It has struck down state attempts to prevent a criminal defendant from calling an accomplice as a witness in his favor,^151^ bar a defendant from impeaching his own witness,^152^ and bar a criminal defendant from questioning the prosecution’s star witness about the latter’s juvenile criminal record.^153^

Moreover, privileges that have been held to give way under some circumstances include physician–patient, psychotherapist–patient, rape counselor, spousal, and marriage counselor–client privileges.^154^ Courts have held that even the attorney–client privilege must ‘yield when the accused establishes an exceptionally strong need for the privileged information’.^155^ As one recent case summarized, ‘the majority of jurisdictions in the United States have determined that a criminal defendant’s right, provided certain requirements are met, may supersede a witness’s rights or statutory privilege’.^156^

This does not mean, however, that privileges give way routinely or easily in the face of constitutional criminal defense rights. Even among courts that have recognized that a defendant’s constitutional rights may trump a witness’s privilege, courts have demanded an exacting showing of need by the defendant.^157^ For example, courts have quashed defense subpoenas for privileged records where the court concluded that the defense was engaged in a ‘broad “fishing expedition.”’^158^ Where defendants ‘had only speculated’ or made ‘vague assertions’ that the records sought could contain material exculpatory or impeaching evidence, courts have rejected any right of access.^159^ Courts have also denied access to privileged information when it is available from other sources.^160^ In other words, courts do not supersede privilege lightly, and they have demanded substantial showings of likely relevance and materiality before requiring any form of disclosure for otherwise privileged records.

The Supreme Court has also held that a criminal defendant’s constitutional rights could be safeguarded by a trial court’s *in camera* review of otherwise confidential records.^161^ Thus, even if a defendant has surmounted this exacting standard to obtain some form of access to otherwise privileged records, the court will review the records itself and disclose to defense counsel only the subset of records, if any, that are genuinely relevant and material to the defense. Only in rare instances have courts held that defense counsel is entitled to full review of otherwise-privileged records.^162^

It is conceivable that, in a criminal case, a witness’s statements in the context of a research study could be so singularly relevant and material either to impeaching the witness or exculpating the defendant that privilege must give way. But this is likely to occur with exceptional infrequency. As the appellate court in *North Carolina v Bradley* explained, ‘just because defendant asks for an *in camera* inspection does not automatically entitle him to one’.^163^ Nonetheless, while *Bradley* grasped that sensitive information was at issue, that court erred in considering materiality before privilege. Privilege sets a high bar against disclosure, whereas materiality sets a low bar in favor of disclosure. Had the trial court started with the privilege the Certificate represents, it likely would have rejected defense access on that basis, concluding that this was not an exceptional case. At most, it might have ordered *in camera* review of the disputed data, rather than ordering unwarranted disclosure for the appeal. Similarly, had the appellate court started with privilege, it would have more clearly demonstrated the trial court’s error while acknowledging the power of the Certificate’s protections.

Significantly, the fact that criminal defendants may, in exceptional circumstances, gain access to Certificate-protected information does not mean that law enforcement investigators or prosecutors will get access to that information. Although the Supreme Court has recognized that defense discovery rights are at least as broad as those granted to the State, the opposite is not true.^164^ Indeed, the Court has observed that ‘if there is to be any imbalance in discovery rights, it should work in the defendant’s favor’.^165^ This imbalance is all the more sensible given that the original Certificate statute was enacted in response to concerns about law enforcement access to identifiable, sensitive research data related to drug abuse.^166^

In sum, while it is unlikely that constitutional criminal defense rights can *never* overcome the privilege the Certificate accords, cases in which the Certificate’s protections will be overcome will be few and far between. Courts have imposed exacting standards even in criminal cases for displacing privilege, and even when those standards have been met, the requisite remedy has largely been limited to *in camera* review.

## IV. IMPLICATIONS

Our analysis demonstrates that Certificates fit squarely within Congress’s constitutional authority under the Commerce Clause and Spending Power. This is true whether we consider Congress’s authority to regulate ‘persons or things in interstate commerce’ or its authority to regulate ‘activities having a substantial relation to interstate commerce’. That Certificates were adopted—and amended—as part of a comprehensive legislative scheme lends additional support to this conclusion. Congress has amended the Certificate-authorizing statute multiple times over 50 years to extend the protections and, most recently, strengthen them. This history demonstrates both Congress’s belief in its authority and that Congress values this protection. Our analysis and its conclusions should provide welcome reassurance to researchers and IRBs who rely on Certificates to protect participants’ research data. They can have confidence in the promises they make to participants during the informed consent process.

An oft expressed concern about Certificates is whether their protections would fall if a criminal defendant were to seek access to protected research data. Our analysis should provide reassurance on this point as well. While our analysis of the law of privileges suggests that courts *may* sometimes order disclosure of information protected by a privilege, those cases are rare and require exacting proof. Such exceptions are recognized as precisely that extraordinary exceptions to the privilege that do not negate the existence of the privilege itself. Thus, even if, in an extraordinary case, protected research data are ordered revealed, the Certificate statute will survive to protect other research data. Moreover, given that the Certificate statutory language is more explicit and stronger than other federal privileges that have repeatedly been upheld, it is likely that these exceptional cases will be very rare.

Judges and legal counsel often are unfamiliar with the protections offered by Certificates.^167^ For example, the *Bradley* court relied on the more familiar concept of materiality, rather than the Certificate, in determining whether the defendant should have access to protected research data. Other courts and legal counsel have referred to Certificates in circumstances where the protections do not exist. Given that many institutional counsel have limited experience with demands for research data, it is understandable that Certificates are unfamiliar to many.^168^ But lack of familiarity can also lead to disclosures that break promises to research participants and undermine public trust in research while also subjecting researchers and institutions to penalties for failing to comply with a grant’s terms and conditions. We hope that our analysis equips all stakeholders to implement Certificates’ protections appropriately and vigorously.

Our insights about how Certificates fit within the broader law of statutory privileges may also provide a mechanism for educating lawyers and judges about their protections. Privilege is a familiar concept to most lawyers. Organizations that provide education and other resources for judges (eg Federal Judicial Center, Appellate Judges Education Center) and institutional legal counsel (eg National Association of University and College Attorneys) can and should include information about Certificates as a privilege in their courses and materials. We think that including the appropriate framework for thinking through exceptional cases—starting with the privilege—in educational materials could help avoid some of the problems raised by the *Bradley* case.

To fulfill researchers’ ethical commitments to participants reflected in Certificates, additional education may also be needed. There is a continuing need to educate IRBs, researchers, and research participants about Certificates to enable them to make informed decisions. Previous research demonstrated misunderstanding about Certificates before the 21st Century Cures Act Amendments.^169^ At that time, researchers had to apply for a Certificate, whether prompted by their own concern for participant confidentiality or required by their IRB.^170^ Accordingly, those researchers knew about the obligations they had undertaken and could assert them. Under 21st Century Cures Act Amendments and automatic issuance of Certificates for NIH-funded research, it is not clear the extent to which researchers are even aware of and understand those protections and obligations. Federal departments and agencies may need additional information as well. Although not the only agency to issue Certificates, NIH has long taken the lead in providing information about Certificates through its website and through educational programs, such as in presentations at the Public Responsibility in Medicine & Research annual conference.^171^ NIH could help other agencies and their grantees make full use of this tool now that the Secretary must issue a Certificate to all federally funded research when requested.

Finally, the federal government should provide support when researchers and institutions must assert Certificates’ protections in court. Having made the issuance of Certificates automatic for the human subjects research it funds and incorporated its requirements into the terms and conditions of funded grants, the federal government has an obligation to assist parties to enforce these protections.

We do not expect the federal government to take on legal representation in individual cases, but it could help attorneys find the information they need to use Certificates to protect research data. Currently, the only case to which the NIH Certificate website refers is *People v Newman*.^172^ But *Newman* does not tell the full tale of Certificates in courts. Directing counsel (and judges) to more complete information could provide the background necessary to advance the legal arguments necessary to maintain the confidentiality of protected data and avoid unauthorized and unwarranted disclosures that could give rise to penalties. Additionally, the Secretary of HHS or a designee could provide an affidavit that outlines the legal authority for the Certificate and the protections it affords to recipients facing a legal demand. Such an action would be consistent with the federal government’s expansion of the use of Certificates reflected in the 21st Century Cures Act amendments mandating issue of a Certificate to federally funded research, as well as NIH’s decision to issue Certificate automatically protecting the research it funds, making compliance part of the terms and conditions of grants, and imposition of penalties for impermissible disclosures. An affidavit from the responsible federal official would buttress the case that such protections exist.

## V. CONCLUSION

Over 50 years ago, Congress recognized that protecting the confidentiality of identifiable research data was essential to our ability to conduct high-quality biomedical research on sensitive topics that improves the health and well-being of all. It has reiterated that commitment multiple times, as it has expanded the scope of Certificates. Researchers, IRBs, and research participants have relied on those protections to advance important research, but concerns about their legal effectiveness have influenced how strongly Certificates have been asserted to protect data in the face of a legal demand. Our analysis provides important reassurance that Certificates afford robust protection while also providing practical suggestions for ensuring enforcement of those protections.

## Footnotes

1 Dobbs v. Jackson Women’s Health Organization, No. 19-1392, 597 U.S. __ (June 24, 2022).

2 Lisa H. Harris, *Navigating Loss of Abortion Services—A Large Academic Medical Center Prepares for the Overturn of* Roe v. Wade, 386 NEJM 2061 (2022); I. Glenn Cohen, Judith Daar & Eli Y. Adashi, *What Overturning* Roe v. Wade *May Mean for Assisted Reproductive Technologies in the US,* 328 JAMA 15 (2022); I. Glenn Cohen, Melissa Murray & Lawrence O. Gostin, *The End of* Roe v. Wade *and New Legal Frontiers on the Constitutional Right to Abortion*, 328 JAMA 325 (2022).

3 Kayte Spector-Bagdady & Michelle M. Mello, *Protecting the Privacy of Reproductive Health Information After the Fall of* Roe v. Wade, 3 JAMA Health  Forum e222656 (2022); Tracey A. Wilkinson, *Dr Caitlin Bernard Was Meant to Write This With Me Before She Was Attacked for Doing Her Job*, N.Y. Times, July 15, 2022.

4 Leslie E. Wolf and Jolanta Zandecki, *Sleeping Better at Night: Investigators’ Experiences With Certificates of Confidentiality*, 28 IRB: Ethics & Human  Research 1 (2006).

5 People v. Newman, 32 N.Y.2d 379 (1973) (photos of methadone maintenance clinic patients subpoenaed for use in identifying a murderer; Certificate prevented production).

6 People v. Still, 369 N.Y.S.2d 759 (App. Div 1975) (defendant sought release of his own clinic records from methadone maintenance clinic to demonstrate he possessed methadone lawfully; production permitted based on defendant-patient’s consent); Bradley v. North Carolina, 634 S.E.2d 258 (N.C. Ct. App. 2006) (defendant sought research data to impeach witness; data ultimately determined to be immaterial to defendant’s case and withheld on those grounds, rather than on the Certificate’s protection).

7 Order re Motion to Quash Subpoenas Re Yale Study’s Hospital Records, *In re* Phenylpropanolamine (PPA) Products Liability Litigation, No. 1407 (W.D. Wash. Aug. 19, 2002); Confidentiality Order re WHI Study Data, PremPro Products Liability Litigation No. 4:03-CV-01507-WRW (E.D. Ark. Feb. 1, 2005); Dummit v. CSX Transport, Inc., No. 01-C-145 (Cir. Ct. W. Va. Nov. 21, 2006) (on file with authors).

8 Louisville Branch—Nat’l Ass’n for the Advancement of Colored People, 06-ORD-094, Op. Ky. Att’y Gen. (2006) (refusing access to data from study evaluating racial fairness in sentencing).

9 Memorandum of Decision on Motion to Quash, Connecticut Superior Court for Juvenile Matters (Jud. Dist. Hartford July 1, 2003) (granting state Department of Children and Families access to research data despite Certificate’s protections).

10 Natalie Ram, et al., *Genealogy Databases and the Future of Criminal Investigations*, 360 Science 1078 (2018); Virginia Hughes, *To Solve 3 Cold Cases, This Small County Got a DNA Crash Course,* N.Y. Times, May 3, 2021; Raffi Khatchadourian, *How Your Family Tree Could Catch a Killer*, New  Yorker, Nov. 15, 2021; Leslie E. Wolf and Laura M. Beskow, *Genomic Databases, Subpoenas, and Certificates of Confidentiality*, 21 Genetics in  Medicine 2681 (2019).

11 Natalie Ram, *America’s Hidden National DNA Database*, 100 Tex. L. Rev. 1253 (2022); Emily Mullin, *Police Used a Baby’s DNA to Investigate Its Father for a Crime*, Wired, Aug. 15, 2022; Dana Difilippo, *Judge Orders State to Release Information About Police Use of Baby Blood Spots*, N. J. Monitor, Jan. 4, 2023.

12 45 C.F.R. 46.111(a)(1) & (2) (2018); Leslie E. Wolf et al., *Certificates of Confidentiality: Protecting Human Subject Research Data in Law and Practice*, 14 Minn. J. L. Sci. & Tech. 11, 18–20 (2013).

13 NIH, *Certificate of Confidentiality (CoC)*, https://grants.nih.gov/policy/humansubjects/coc.htm (last accessed Feb. 7, 2024).

14 NIH, *Certificate of Confidentiality (CoC)*, https://grants.nih.gov/policy/humansubjects/coc.htm (last accessed Feb. 7, 2024).

15 42 U.S.C. § 241(d)(1)(A).

16 NIH, *Notice of Changes to NIH Policy for Issuing Certificates of Confidentiality, NOT-OD-17-109*, Sept. 7, 2017, https://grants.nih.gov/grants/guide/notice-files/NOT-OD-17-109.html (last accessed Feb. 7, 2024).

17 See Wolf and Zandecki, *supra* note 4, at 3–4; Laura M. Beskow, et al., *Institutional Review Boards’ Use and Understanding of Certificates of Confidentiality*, 7 PLoS One 7 at 3, 344050 (2012).

18 See Wolf and Zandecki, *supra* note 4, at 3–4; Beskow et al. (2012), *supra* note 17, at 3.

19 See Wolf and Zandecki, *supra* note 4, at 5; Beskow et al. (2012), *supra* note 17, at 5–8; Leslie E. Wolf et al., *Certificates of Confidentiality: Legal Counsels’ Experiences With and Perspectives on Legal Demands for Research Data*, 7 J. Empirical  Res. on  Hum. Subjects  Res. 1, 5 (2012).

20 Wolf et al. (2012), *supra* note 19, at 5.

21 Natalie Ram, Jorge Contreras, Laura M. Beskow, and Leslie E. Wolf, *Constitutional Confidentiality*, 80 Wash. & Lee L. Rev. 1349 (2023).

22 Comprehensive Drug Abuse Prevention and Control Act of 1970, Pub. L. No. 91–513, 84 Stat. 1236, 1241 (1970); Wolf et al. (2013), *supra* note 12.

23 H.R. Rep. No. 91–1444 (1970), reprinted in 1970 U.S.C.C.A.N. 4566, 4567.

24 Wolf et al. (2013), *supra* note 12, at 21–22.

25 Wolf et al. (2013), *supra* note 12, at 22 (researcher and subject ‘agreed that [they] would face contempt rather than betray a confidence’ and the confidentiality protections ‘will permit field investigations to get information the public really wants without turning research investigators into [. . .] criminals if they ethically insist on withholding personal information.’) (citing to Comprehensive Narcotic Legislation: Hearings on S. 1895 m S. 2590, H.R. 10,019, and S. 2637 Before the Subcomm. To Investigate Juvenile Deliquency, S. Comm. On the Judiciary, 91st Cong. 315–321 (1969).)

26 42 U.S.C. § 241(d)(1)(D).

27 The Health Omnibus Programs Extension of 1988, Pub. L. 100–607, 102 Stat. 3048 (1988), 42 U.S.C.. § 241 (d)(1)(A) (2018).

28 NIH, *Mission and Goals*, https://www.nih.gov/about-nih/what-we-do/mission-goals (last accessed Feb. 7, 2024).

29 Leslie E. Wolf & Laura M. Beskow, *New and Improved? 21st Century Cures Act Revisions to Certificates of Confidentiality*, 44 Am. J. Law & Med. 343, 347–353 (2018).

30
*Id*.

31 42 U.S.C. § 241(d)(1)(A) (2018).

32 42 U.S.C. § 241(d)(1)(A)(i) & (ii) (2018).

33 42 U.S.C. § 241(d)(1)(D).

34 42 U.S.C. § 241(d)(1)(D).

35 42 U.S.C. § 241(d)(1)(E).

36 42 U.S.C. § 241(d(1)(F).

37 NIH, *Frequently Asked Questions, Certificates of Confidentiality,* (B.15.), https://grants.nih.gov/faqs#/certificates-of-confidentiality.htm?anchor=55527 (last accessed Feb. 7, 2024).

38 See *discussion* in Wolf et al. (2013), *supra* note 12, at 27–47.

39
*People v Newman*, 32 N.Y.2d 379 (1973).

40
*Id.* at 382.

41
*Id.* at 392.

42
*People v Still*, 369 N.Y.S.2d 759 (App. Div. 1975).

43
*North Carolina v Bradley*, 634 S.E.2d 258 (N.C. Ct. App. 2006).

44
*North Carolina v Bradley* at 261; Laura M. Beskow, Lauren Dame, E. Jane Costello, *Certificates of Confidentiality and the Compelled Disclosure of Research Data*, 322 Science 1054, 1054 (2008).

45 Beskow et al. (2008), *supra* note 44, at 1055.

46 Order Re: Motion to Quash Subpoenas Re Yale Study’s Hospital Records, In re Phenylpropanolamine (PPA) Products Liability Litigation, No. 1407 (W.D. Wash. Aug. 19, 2002); Confidentiality Order Re WHI Study Data, In re PremPro Products Liability Litigation, No. 4:03-CV-01507-WRW (E.D. Ark. Feb. 1, 2005); Dummit v. CSX Transport, Inc., No. 01-C-145 (Cir. Ct. W. Va. Nov. 21, 2006).

47 Memorandum of Decision on Motion to Quash, Connecticut Superior Court for Juvenile Matters (Jud. Dist. Hartford July 1, 2003).

48 Louisville Branch—Nat’l Ass’n for the Advancement of Colored People, 06-ORD-094 Op. Ky. Att’y Gen. (2006).

49 Murphy v. Phillip Morris, No. CV 99–7155-RAP (JWJx) (C.D. Cal. Mar. 17, 2000); Uhr v. University of Minnesota, 2018 WL 414296 (2018); NC Swine Farm Nuisance Litigation, 2016 WL 3742135 (2016); Environmental Defense Fund v. U.S. Environmental Protection Agency, 515 F. Supp. 3d 1135 (2021). See Patterson v. Crowder, 2021 WL 1894152 (2021) (claim by pro se prisoner litigant that includes § 1983 claim); Cantu v. City of Dothan, Alabama, 974 F.3d 1217 (2020) (estate’s assertion of § 1983 claim against officer who shot the decedent during an encounter); Ferreira v. Corsini, 2016 WL 11372329 (2016) (pro se plaintiff with § 1983 claim); Wasseff v. National Institute of Health, 2017 WL 495795 (2017) (former postdoc asserts employment related claims).

50 Wolf et al. (2012), *supra* note 19, at 3–4.

51
*Id.* at 3.

52
*Id*.

53
*Id*.

54 Beskow et al. (2008), *supra* note 44, at 1055.

55 Wolf et al. (2012), *supra* note 19, at 4 & 7; Beskow et al. (2012), *supra* note 17, at 3–4.

56 Ram et al., *supra* note 21.

57 U.S. Const., Article 1, Section 8.

58 United States v. Lopez, 514 U.S. 549, 558–59 (1995).

59 Lopez, 514 U.S. at 559 (emphasis added).

60 United States v. Morrison, 529 U.S*.* 598, 610–612 (statute created a private right of action for gender-motivated violence). See also Lopez, 514 U.S. at 563 (statute sought to regulate (as the court described it) ‘possession of a firearm in a local school zone’).

61 See Wickard v. Filburn, 317 U.S. 111 (1942), Raich v. Gonzales, 545 U.S. 1 (2005).

62 Raich v. Gonzales, 545 U.S. at 17.

63 See NFIB v. Sebelius, 567 U.S. 519, 549–57 (2012) (declining to uphold the Affordable Care Act’s individual mandate provision under the Commerce Clause).

64 U.S. Const. Art. I, § 8, cl. 18.

65 United States v. Lopez, 514 U.S. at 558–559.

66 45 C.F.R. § 46.102(l); see also NIH, Mission and Goals, *supra* note 29.

67 U.S. Food & Drug Administration, *FDA History*, https://www.fda.gov/about-fda/fda-history (last accessed Feb. 7, 2024); Suzanne White Junod, *FDA and Clinical Drug Trials, A Short History*, https://www.fda.gov/media/110437/download (last accessed Feb. 7, 2024).

68 Benjamin Djulbegovic & Gordon H. Guyatt, *Progress in Evidence-based Medicine: A Quarter Century On,* 390 Lancet 415 (2017); Inst. of  Med., The CTSA Program at NIH: Opportunities for  Advancing  Clinical and  Translational  Research, Chapter 2 (2013).

69 Centers for Medicare & Medicaid Services, *NHE Fact Sheet,*  https://www.cms.gov/Research-Statustics-Data-and-Systems/Statistics-Trends-and-Reports/NationalHealthExpendData/NHE-Fact-Sheet (last accessed Feb. 7, 2024).

70 Research America!, *Research Funding and Economic Impact by State*, https://perma.cc/C2VG-ZC5G (last accessed Feb. 7, 2024).

71 Research America!, *U.S. Investments in Medical and Health Research and Development*, 2016–2020, https://www.researchamerica.org/wp-content/uploads/2022/09/ResearchAmerica-Investment-Report.Final_.January-2022-1.pdf (last accessed Feb. 7, 2024).

72 NIH, *U.S. Department of Health & Human Services, What We Do, Budget*, https://www.nih.gov/about-nih/what-we-do/budget (last accessed Feb. 7, 2024).

73 NIH, *supra* note 72.

74 Advisory Committee on Human Radiation Experiments, *Final Report, Chapter 14*, https://ehss.energy.gov/ohre/roadmap/achre/chap14_4.html (last accessed Feb. 7, 2024).

75 Congressionally Directed Medical Research Program, *Department of Defense, 2020 Annual Report*, https://cdmrp.health.mil/pubs/annreports/2020annrep/2020annreport.pdf (last accessed Feb. 7, 2024).

76 PhRMA, *Research & Development Policy Framework*, https://phrma.org/policy-issues/Research-and-Development-Policy-Framework (last accessed Feb. 7, 2024).

77 U.S. Bureau of Labor Statistics, *May 2020 National Industry-Specific Occupational Employment and Wage Estimates, NAICS 325400—Pharmaceutical and Medicine Manufacturing*, https://www.bls.gov/oes/2020/may/naics4_325400.htm (last accessed Feb. 7, 2024); U.S. Bureau of Labor Statistics, May 2020 National Industry-Specific Occupational Employment and Wage Estimates, NAICS 339100—Medical Equipment and Supplies Manufacturing, https://www.bls.gov/oes/2020/may/oessrci.htm (last accessed Feb. 7, 2024); *Biopharmaeutical Spotlight: The Biopharmaceutical Industry in the United States, Biopharmaceuticals Industry*, https://www.trade.gov/selectusa-biopharmaceuticals-industry (last accessed Feb. 7, 2024).

78 Centers for Medicare & Medicaid Services, *supra* note 69; Alex Brill & Benedic Ippolito, *The Economics of Biologic Drugs: A Further Response to Bach et al.*, Health  Affairs  Forefront, Aug. 8, 2019, https://www.healthaffairs.org/content/forefront/economics-biologic-drugs-further-response-bach-et-al (Feb. 7, 2024); Gerald F. Donahoe, Advanced  Medical  Technology  Association, Estimates of  Medical device spending in the united states (2021), https://www.advamed.org/wp-content/uploads/2021/12/Estimates-Medical-Device-Spending-United-States-Report-2021.pdf (last accessed Feb. 7, 2024).

79 Centers for Medicare & Medicaid Services, *Historical*, https://www.cms.gov/Research-Statistics-Data-and-Systems/Statistics-Trends-and-Reports/NationalHealthExpendData/NationalHealthAccountsHistorical#:~:text=U.S.%20health%20care%20spending%20grew (last accessed Feb. 7, 2024). Anne B. Martin, et al., *National Health Care Spending in 2019: Steady Growth for the Fourth Consecutive Year*, 40 Health  Affairs 14 (2021).

80 Christine Grady, *Institutional Review Boards*, 148 Chest 1148, 1150 (2015).

81 NIH, *U.S. Department of HHS, Final NIH Policy on the Use of Single Institutional Review Board for Multi-Site Research, NOT-OD-16-094*, June 21, 2016, https://grants.nih.gov/grants/guide/notice-files/NOT-OD-16-094.html (last accessed Feb. 7, 2024); 45 C.F.R. §46.114.

82 Holly A. Massett et al., *Meeting the Challenge: The National Cancer Institute’s Central Institutional Review Board for Multi-Site Research,* 36 J. Clin. Oncology 819, 820–821 (2018). SMART IRB, a platform for coordinating single IRB review, boards 992 participating institutions (as of June 2022). SMART IRB, https://smartirb.org/about-us/ (last accessed Feb. 7, 2024).

83 JAMA, *For Authors*, https://jamanetwork.com/journals/jama/pages/for-authors (last accessed Feb. 7, 2024).

84 Office of NIH History & Stetten Museum, *National Institutes of Health, A Short History of the National Institutes of Health*, https://history.nih.gov/display/history/A+Short+History+of+the+National+Institutes+of+Health (last accessed Feb. 7, 2024).

85 The National Research Act, Pub. L. 93–348, 88 Stat. 342 (1974).

86
*Id.* § 101.

87 Centers for Disease Control and Prevention, U.S. Department of Health & Human Services, *The Tuskegee Timeline*, https://www.cdc.gov/tuskegee/timeline.htm (last accessed Feb. 7, 2024).

88 The National Research Act, *supra* note 85, §§ 201 et seq.

89
Nat’l  Comm’n for the  Protection of  Human  Subjects of  Biomedical & Behavioral  Res., The  Belmont  Report (1979).

90 45 C.F.R 46 (HHS) (see 46 FR 8366) and 21 C.F.R. 50 & 56 (FDA) (see 46 FR 8942 and 46 FR 8958); Office for Human Research Protections, *Federal Policy for the Protection of Human Subjects (‘Common Rule’)*, https://www.hhs.gov/ohrp/regulations-and-policy/regulations/common-rule/index.html (last accessed Feb. 7, 2024).

91 Food and Drug Administration, *FDA Fundamentals*, https://www.fda.gov/about-fda/fda-basics/fda-fundamentals (last accessed Feb. 7, 2024).

92 United States v. Lopez, 514 U.S. 549, 558 (1995).

93 42 U.S. Code §241(d).

94 United States v. Morrison, 529 U.S. 598, 608–609 (2000).

95 Lopez, 514 U.S. at 558.

96 See NIH, *NIH Grants Policy Statement (2021)*, https://grants.nih.gov/grants/policy/nihgps/HTML5/section_4/4.1.4_confidentiality.htm#Confidentiality (last accessed Feb. 7, 2024).

97
*Contrast* United States v. Lopez, 514 U.S. 549.

98 Morrison, 529 U.S. at 612–613.

99 Morrison, 529 U.S. at 612–613 (internal quotation marks omitted).

100 See NFIB v. Sebelius, 567 U.S. 519, 549 (2012) (quoting Free Enter. Fund v. Pub. Co. Accounting Oversight Bd., 561 U.S. 477, 505 (2010)) (alterations in original); see also *NFIB v OSHA*, 142 S. Ct. 661, 662 (2022) (‘OSHA has never before imposed such a mandate. Nor has Congress.’); Terkel v. CDC, 521 F. Supp. 3d 662, 675 (2021) (holding that the CDC eviction moratorium exceeded Congress’s Commerce Clause authority, and observing as part of its ‘attenuation’ analysis that ‘the federal government has never before invoked its commerce power to impose a nationwide eviction moratorium. Nor has the court’s attention been called to a longstanding analogous use of federal power.’).

101 See, eg Pierce County v. Guillen, 537 U.S. 129 (2003).

102 42 U.S. Code §241(d)(1)(E).

103 Rebecca Wexler, *Privacy as Privilege: The Stored Communications Act and Internet Evidence*, 134 Harv. L. Rev. 2721, 2745–46 (2021).

104 Mila Sohoni, *The Power to Privilege*, 163 U. Pa. L. Rev. 487, 532 (2015).

105 Baldrige v. Shapiro, 455 U.S. 345, 360 (1982) (‘It is well recognized that a privilege may be created by statute,’ in assessing statutory privileges in the Census Act); FRE 501 (stating that judicial common law authority over privilege claims do not apply if a federal statute ‘provides otherwise.’).

106 Wexler, *supra* note 103, at 2748.

107
*Id*.

108
*Id*. at 2748–2749.

109 Pierce County. v. Guillen, 537 U.S. 129, 144 (2003).

110 See St. Regis Paper Co. v. United States, 368 U.S. 208, 218 (1961) (declining to find a statutory privilege).

111 Baldrige v. Shapiro, 455 U.S. 345, 360 (1982) (quoting St. Regis Paper, 368 U.S. at 218).

112 St. Regis Paper Co. v. United States, 368 U.S. 208, 218 (1961) (analyzing 13 U.S.C. § 9(a) (1958) (current version at 13 U.S.C. § 9(a))).

113 13 U.S.C. § 9(a) (as amended on Oct. 15, 1962, Pub.L. 87–813, 76 Stat. 922).

114 Baldrige v. Shapiro, 455 U.S. 345, 362 (1982).

115 Baldrige v. Shapiro, 455 U.S*.* at 361.

116 537 U.S. 129 (2003).

117
*Id.* at 132.

118
*Id.* at 147.

119 23 U.S.C. § 409.

120 Wexler, *supra* note 103, at 2763–2764; Jicarilla Apache Nation v. United States, 60 Fed. Cl. 611, 612 (2004).

121 Wexler, *supra* note 103, at 2763–2764.

122 See St. Regis Paper Co. v. United States, 368 U.S. 208, 218 (1961); Jicarilla Apache Nation, 60 Fed. Cl. at 612; Wexler, *supra* note 103.

123 42 U.S.C. § 241(d)(1)(E).

124 42 U.S.C. § 241(d)(1)(E).

125 42 U.S.C. § 241(d)(1)(D).

126 42 U.S.C. § 241(d)(1)(F).

127 Guillen, 537 U.S. at 147.

128 See, eg 13 U.S.C. § 9(a).

129 See, eg 42 U.S.C. § 10,708(b); 42 U.S.C. § 3789 g(a). Ram et al., *supra* note 21.

130 18 U.S.C. § 2701–2712.

131 Gonzales v. Raich, 545 U.S. 1, 23 (2005).

132
*Id*. at 32.

133
*Id.* at 24.

134
*Id.* at 37 (Scalia, J., concurring).

135
*Id.* at 15.

136 See Pub. L. 91–513, Title I, Section 3(a), 84 Stat. 1241 (1970).

137 Public Law 114–255, 130 Stat.1033 (2016).

138
*Gonzales v Raich*, *supra* note 131.

139 Ram et al., *supra* note 21.

140 NFIB v. Sebelius, 567 U.S. at 536 (2012).

141 Matthew D. Adler, *State Sovereignty and the Anti-Commandeering Cases*, 574 Annals  Am. Acad. Political & Social  Sci. 158, 158 (2001).

142 Printz v. United States, 521 U.S. 898 (1997).

143 Murphy v. NCAA*,* 138 S. Ct. 1461 (2018).

144 Reno v. Condon, 528 U.S. 141 (2000).

145 Reno v. Condon, 528 U.S. 141 (2000).

146 Ram et al., *supra* note 21.

147 See Pierce Cty. v. Guillen, 537 U.S. 129,148 n.10 (2003) (explicitly reserving the question of whether such a privilege ‘violates the principles of dual sovereignty embodies in the Tenth Amendment because it prohibits a State from exercising its sovereign powers to establish discovery and admissibility rules to be used in state court for a state cause of action.’).

148 Haaland v. Brackeen, 143 S. Ct. 1609, 1635 (2023); New York v. United States, 505 U.S. 144, 178–179 (1992). Adam B. Cox, *Expressivism in Federalism: A New Defense of the Anti-Commandeering Rule?*, 33 Loy. L.A. L. Rev. 1309, 1310 (2000).

149 Wolf et al. (2012) *supra* note 19, at 5; Wolf et al. (2013), *supra* note 12, at 48.

150 See Holmes v. South Carolina, 547 U.S. 319, 324 (2006) (‘Whether rooted directly in the Due Process Clause of the Fourteenth Amendment or in the Compulsory Process or Confrontation Clauses of the Sixth Amendment, the Constitution guarantees criminal defendants a meaningful opportunity to present a complete defense.’) (quotation marks omitted).

151 Washington v. Texas, 388 U.S. 14 (1967).

152 Chambers v. Mississippi, 410 U.S. 284, 302 (1973).

153 Davis v. Alaska, 415 U.S. 308, 318–320 (1974).

154 Edward J. Imwinkelried, *Questioning the Behavioral Assumption Underlying Wigmorean Absolutism in the Law of Evidentiary Privileges*, 65 U. Pitt. L. Rev. 145, 167 (2004).; see also Christopher B. Mueller & Laird C. Kirkpatrick, 2 Federal  Evidence § 5:10 (4th ed.).

155 Imwinkelried, *supra* note 154, at 167; Vela v. Superior Court, 255 Cal. Rptr. 921 (1989); and State v. Pearson, 22 A.T.L.A. L. Rep. 409 (Ohio Ct. App. 1979).

156 State v. Blackwell, 801 S.E.2d 713, 726 (S.C. S. Ct. 2017) (collecting cases).

157 Imwinkelried, *supra* note 154.

158 State v. Olah, 184 A.3d 360, 371 (Me. 2018).

159 State v. Olah, 184 A.3d 360, 371 (Me. 2018); see also People v. Foggy, 521 N.E.2d 86, 92 (Ill. 1988); People v. District Court, 719 P.2d 722, 726 (Colo. 1986).

160 Foggy, 521 N.E.2d at 91.

161 Pennsylvania v. Ritchie, 480 U.S. 39, 61 (1987). See also Blackwell, 801 S.E.2d at 726, 728; see also State v. Olah, 184 A.3d 360, 369 (Me. 2018).

162 See Com. v. Dwyer, 859 N.E.2d 400, 419 (Mass. 2006) (‘all presumptively privileged records that are summonsed shall be retained in court under seal, and shall be inspected only by counsel of record for the defendant who summonsed the records.’).

163 North Carolina v. Bradley, 634 S.E.2d 258, 262 (N.C. Ct. App. 2006).

164 Wardius v. Oregon, 412 U.S. 470, 471–472 (1973); see also *Williams v Florida*, 399 U.S. 78 (1970).

165 Wardius, 412 U.S. at 475 n. 9.

166 Wolf et al. (2013), *supra* note 12, at 21–24.

167 Wolf et al. (2012), *supra* note 19, at 7; Wolf et al. (2013), *supra* note 12, at 48 & 86.

168 Wolf et al. (2012), *supra* note 19, at 7.

169 Beskow et al. (2012), *supra* note 17, at 3–4; Wolf et al. (2012), *supra* note 19, at 7.

170 Wolf and Zandecki, *supra* note 4, at 4.

171 U.S. Department of HHS, NIH, *Certificates of Confidentiality*, https://grants.nih.gov/policy/humansubjects/coc.htm (last accessed Feb. 7, 2024); Sarah Luery, *Adapting Certificates of Confidentiality Policy Under the 21st Century Cures Act: Benefits and Challenges*, Apr. 5, 2018 https://blog.primr.org/adapting-certificates-of-confidentiality-policy-under-the-21st-century-cures-act-benefits-and-challenges/ (last accessed Feb. 7, 2024) (describing session at 2017 PRIMR conference, including Petrice Brown-Longenecker and Elonna Ekweani from NIH).

172 NIH, *Frequently Asked Questions, Certificates of Confidentiality, Legal Considerations* (F.1.), https://grants.nih.gov/faqs#/certificates-of-confidentiality.htm?anchor=50090 (last accessed Feb. 7, 2024).

